# Effects of Heat Stress on Semen Quality and Reproductive Performance of Crossbred Boars with Different Maternal Origins

**DOI:** 10.3390/ani16071080

**Published:** 2026-04-01

**Authors:** Hsiu-Lan Lee, Ting-Yung Kuo, Lin-Liang Peng, Chin-Bin Hsu, Cheng-Yung Lin, Shen-Chang Chang, Yen-Hua Lee, I-Ling Lai, Hsiu-Luan Chang, Perng-Chih Shen

**Affiliations:** 1 Graduate Institute of Bioresources, National Ping Tung University of Science and Technology, Pingtung 91201, Taiwan; hlli@tlri.gov.tw (H.-L.L.); ilai@mail.npust.edu.tw (I.-L.L.); 2 Technical Service Division, Taiwan Livestock Research Institute, Ministry of Agriculture, Tainan 71246, Taiwan; jengyong@tlri.gov.tw; 3 Genetics and Physiology Division, Taiwan Livestock Research Institute, Ministry of Agriculture, Tainan 71246, Taiwan; tykuo@mail.tlri.gov.tw (T.-Y.K.); liang0015@tlri.gov.tw (L.-L.P.); 4 Animal Nutrition Division, Taiwan Livestock Research Institute, Ministry of Agriculture, Tainan 71246, Taiwan; cbhsu@mail.tlri.gov.tw; 5 Department of Animal Science, National Chiayi University, Chiayi 60004, Taiwan; schang6161@mail.ncyu.edu.tw; 6 Department of Animal Science, National Pingtung University of Science and Technology, Pingtung 91201, Taiwan; yhlee@mail.npust.edu.tw

**Keywords:** Duroc, Meishan, semen, maternal inheritance, reproductive performance

## Abstract

This study investigated whether maternal origin affects boar semen thermotolerance and its seasonal reproductive performance. Crossbred boars with different maternal origins were evaluated (K and KD boars with Meishan-derived cytoplasm and DK boars with Duroc-derived cytoplasm). Heat stress treatment (42 and 39 °C) on semen samples significantly reduced sperm viability and acrosome/membrane integrity while increasing mitochondrial damage. Notably, the decline in semen quality under heat stress was significantly lower (*p* < 0.05) in K and KD boars than in DK boars. The farrowing rates during the hot season were significantly higher (*p* < 0.05) in the K and KD breed compared with the DK breed. No significant differences among groups were observed during the cool season. Collectively, these findings indicate that maternal origin may contribute to mitochondrial stability and semen resilience under tropical heat stress, thereby influencing reproductive performance in hot seasons. Maternal inheritance should therefore be considered in breeding strategies of improving heat tolerance in pigs.

## Simple Summary

Rising global temperatures pose a serious challenge to pig production, particularly in tropical and subtropical regions. Heat stress can reduce semen quality in boars and decrease reproductive performance in sows, leading to economic losses. In this study, we investigated whether the maternal origin of mitochondria (cytoplasmic inheritance) influences heat tolerance in hybrid boars. We compared hybrid pigs that shared the same nuclear genetic background but differed in maternal cytoplasmic origin, either derived from Meishan or Duroc breeds. Semen samples were incubated at high temperatures in vitro, and reproductive performance was evaluated during the hot and cool seasons. Pigs with Meishan derived cytoplasm showed better semen stability under heat stress and achieved higher farrowing rates during the hot season. These findings suggest that maternal lineage may play a role in improving the heat resilience of pigs. Selecting appropriate maternal lines could therefore be a useful strategy for breeding pigs better adapted to tropical climates.

## 1. Introduction

The major commercial pig breeds currently raised worldwide include Landrace (L), Yorkshire (Y), and Duroc (D). In practical production systems, Landrace or Yorkshire sows, or their F1 crossbred females (LY, YL), are commonly used as maternal lines, whereas Duroc is used as the terminal sire to produce two-way or three-way crossbred market hogs. This crossbreeding strategy aims to combine maternal reproductive capacity with the superior growth rate and carcass traits of the terminal sire. Previous studies have shown that three-way crossbreds or Duroc-terminal cross combinations can improve average daily gain and feed efficiency [1,2,3,4]. In Taiwan, LD and LYD crossbreeding systems remain the dominant hog production models [5].

Comparative studies between European commercial breeds and Chinese indigenous pigs have consistently shown superior growth performance in European breeds [6,7,8]. However, long-term selection strategies primarily targeting growth traits have been associated with unfavorable correlated responses, including deterioration in meat quality [9] and reduced reproductive performance [10,11,12]. In contrast, Meishan pigs have been widely documented to possess exceptional reproductive performance [13,14] and favorable meat quality traits [9]. Consequently, crossbreeding programs have attempted to introduce prolificacy and meat quality characteristics from the Meishan breed into European breeds in order to enhance economic value [9,12,15]. Similarly, in Taiwan, Meishan sows (M) have been crossed with Duroc boars (D) to develop the KHAPS black pig (K), aiming to select a solid black pig breed with a better growth performance while retaining prolificacy [16].

Heat stress induced by elevated ambient temperature negatively affects pig productivity, feed efficiency, reproductive performance, and overall production efficiency, resulting in considerable economic losses to the swine industry worldwide [17]. Previous studies have demonstrated marked seasonal variation in semen quality of major commercial breeds, with superior semen quality observed in cooler seasons compared with hotter seasons [18,19,20]. Such seasonal impairment in semen quality is associated with reduced conception rates in sows [18,21,22]. Moreover, elevated ambient temperatures can disrupt endocrine secretion patterns and ovulatory function in sows, thereby impairing reproductive performance [23,24].

Although indigenous pig breeds originating from tropical and subtropical regions generally exhibit inferior growth performance [8,15], direct comparisons of thermotolerance between these breeds and temperate-origin commercial breeds remain limited [17]. However, *Bos indicus* cattle demonstrate superior heat tolerance and reproductive performance under high-temperature environments compared with *Bos taurus* cattle [25]. At the cellular level, lymphocytes from heat-tolerant breeds (e.g., Brahman) show higher survival rates and lower apoptosis following heat stress exposure [26]. Moreover, in cattle, substantial evidence indicates that thermotolerance may be closely associated with maternal cytoplasmic inheritance [27]. At the embryonic level, embryos derived from heat-tolerant oocytes exhibit higher blastocyst development rates after heat treatment, suggesting that oocyte cytoplasm plays a critical role in thermotolerance [27]. Furthermore, somatic cell nuclear transfer (SCNT) studies in cattle have shown that transferring nuclei from heat-sensitive breeds into oocytes from heat-tolerant breeds improves post-heat-treatment developmental competence, and that this cytoplasm-associated thermotolerance can be transmitted to subsequent generations [28,29,30]. Collectively, these findings suggest that maternal cytoplasmic components can regulate thermotolerance, potentially with transgenerational effects; however, whether similar maternal effects exist in pigs remains unclear.

Sperm motility relies primarily on ATP generated by mitochondrial oxidative phosphorylation, and heat stress is known to induce mitochondrial dysfunction and oxidative damage, thereby impairing semen quality [31,32,33,34]. Because mitochondrial DNA is strictly maternally inherited, maternal cytoplasmic components may play a key regulatory role in maintaining semen stability under heat stress conditions [35].

To study maternal effects in pigs, we designed a reciprocal crossbreeding experiment using KHAPS black pigs (K), which is 100% Meishan cytoplasm and nuclear genome with 50% Meishan and 50% Duroc as the base population [16]. K sows were mated with D boars to produce KD (100% Meishan cytoplasm), whereas D sows were mated with K boars to produce DK (100% Duroc cytoplasm). This design ensured nuclear genetic composition (25% M + 75% D) between KD and DK groups while differing in maternal cytoplasmic origin, thereby allowing separation of cytoplasmic effects from nuclear genetic influences. Seasonal reproductive performance data were collected during cool and hot seasons to further clarify the role of maternal inheritance in thermotolerance.

## 2. Materials and Methods

### 2.1. Animal Ethics

All experimental procedures were reviewed and approved by the Institutional Animal Care and Use Committee (IACUC) of the Kaohsiung Animal Propagation Station, Taiwan Livestock Research Institute, Ministry of Agriculture, Taiwan (approval no. KU 107-04).

### 2.2. Animals and Management

Boars were obtained from the Pingtung facility of the Southern Branch of the Taiwan Livestock Research Institute, Ministry of Agriculture (22.6337° N, 120.6031° E). Boars were trained for dummy-mount semen collection from 7 months of age, and semen was collected every 4–5 days. Boars were housed individually in naturally ventilated conventional barns with ad libitum access to water and were fed 2 kg/day of a corn–soybean meal-based diet containing 16% crude protein. Sows were housed in naturally ventilated conventional barns with ad libitum access to water and were fed 2–3 kg/day of a corn–soybean meal-based diet containing 16% crude protein.

### 2.3. Genetic Composition of Experimental Pigs

The pig breeds used in this study included Meishan (M), Duroc (D), KHAPS black pigs (K), and their reciprocal crossbred groups (KD and DK). The breeding scheme and the proportions of nuclear and cytoplasmic genetic inheritance are summarized in Table 1.

The genetic compositions of the experimental groups were defined according to their pedigree schemes and inheritance patterns. Cytoplasmic inheritance was determined by the maternal lineage, whereas nuclear inheritance was calculated based on pedigree proportion records maintained by Taiwan Livestock Germplasm Information Network (https://www.angrin.tlri.gov.tw/, Livestock Research Institute, Ministry of Agriculture).

Five genetic groups were included:

(1) M: Pure Meishan line; both cytoplasmic and nuclear inheritance were 100% derived from Meishan (M).
(2) D: Pure Duroc line; both cytoplasmic and nuclear inheritance were 100% derived from Duroc (D).
(3) K (M × D): Progeny of M sow × D boar; cytoplasmic inheritance was 100% from M, with nuclear inheritance consisting of 50% M and 50% D.
(4) KD (K × D): Progeny of K sow × D boar; cytoplasmic inheritance remained 100% from M, while nuclear inheritance consisted of 25% M and 75% D.
(5) DK (D × K): Progeny of D sow × K boar; cytoplasmic inheritance was 100% from D, with nuclear inheritance consisting of 25% M and 75% D.

This breeding design enabled the comparison of maternal effects under similar nuclear genetic compositions (e.g., KD vs. DK), thereby allowing evaluation of potential cytoplasmic inheritance influences on semen quality and reproductive performance.

### 2.4. In Vitro Heat Stress Treatment and Semen Quality Assessment

Boars from three genetic groups (K, KD, and DK; 1.0–1.5 years old) were used. To minimize potential confounding effects of seasonal heat stress on baseline semen quality, semen collection was conducted during the cool season (December to March). Semen of four boars per group was collected using the gloved-hand technique with a dummy sow. The penis was rinsed with physiological saline before collection. Ejaculates were collected into a container fitted with a filter to remove the gel fraction. After collection, the gel fraction was removed, and the semen was kept at 37 °C during transport to the laboratory for routine semen evaluation and concentration determination [35].

Semen samples were collected from four boars of each breed over the course of the experimental period. In each trial, one boar per breed was selected for collection, and every boar was sampled on three separate occasions. Immediately after collection, raw semen was extended with a commercial extender (Easy Buffer; IMV Technologies, L’Aigle, France) to achieve a standardized sperm concentration of 7 × 10^7^ cells/mL. For each breed, the extended semen was divided into ten aliquots. One aliquot served as the fresh, unincubated control (0 h). The remaining nine aliquots were assigned to a 3 × 3 factorial arrangement of three storage temperatures and three incubation durations. The three temperature treatments were 42 °C (heat stress), 39 °C (physiological core temperature of the sow), and 17 °C (conventional semen storage temperature). Ali-quots were dispensed into 10 cm culture dishes and placed in a culture chamber maintained at a controlled atmosphere of 5% CO_2_, 5% O_2_, and 90% N_2_. The dishes were then transferred to a programmable temperature-controlled incubator (model CLT-20LB(W); SONGEN, China) set to the designated temperature. At 6, 12, and 24 h of incubation, one set of aliquots from each temperature group was removed for immediate assessment of sperm quality parameters.

Sperm structural and functional integrity were evaluated using a Guava^®^ Easy Cyte micro-capillary flow cytometer (Guava Technologies Inc., Hayward, CA, USA). All assays were performed according to the manufacturer’s instructions (IMV Technologies, L’Aigle, France, https://www.imv-technologies.com/documents/EASYKIT-1-FY-ENG.pdf, accessed on 9 March 2018).

Sperm viability was assessed using the Easy Kit™ Viability kit (cat. no. 024708, IMV Technologies), containing SYBR-14 and propidium iodide (PI), which differentiate live (membrane-intact) from dead (membrane-damaged) spermatozoa. Samples were incubated at 37 °C for 10 min prior to flow cytometric analysis [35].

Acrosome integrity was evaluated using the Easy Kit™ Acrosome Integrity kit (cat. no. 025293, IMV Technologies), containing peanut agglutinin (PNA), SYTO-83™, and PI to simultaneously assess acrosomal disruption in live and dead spermatozoa. Samples were incubated at 37 °C for 45 min in the dark before analysis [35].

Mitochondrial activity and functional integrity were determined using the Easy Kit™ Mitochondrial Activity kit (cat. no. 024864, IMV Technologies), which contains JC-1 dye. JC-1 differentiates polarized mitochondria (orange/red fluorescence) from depolarized mitochondria (green fluorescence). Samples were incubated at 37 °C for 30 min in the dark prior to flow cytometric acquisition. Mitochondrial status was expressed as the percentage of spermatozoa with polarized mitochondria [35].

### 2.5. Reproductive Performance Evaluation

#### 2.5.1. Ambient Temperature, Relative Humidity, and THI

Environmental and reproductive data were collected from June 2019 to March 2024. Hot season data were defined as those recorded from June to September, whereas cool season data were collected from December to March. The temperature–humidity index (THI) was calculated using the equation described in the National Weather Service Central Region report [36]:

$$\mathrm{THI}=(1.8T+32)-\left[0.55\times \left(\frac{RH}{100}\right)\right]\times \left[(1.8T+32)-58\right]$$

where T is ambient temperature (°C), and RH is relative humidity (%). THI categories were defined as: THI < 74 (comfortable), 74 ≤ THI < 78 (mild heat stress), 78 ≤ THI < 82 (moderate heat stress), and THI ≥ 82 (severe heat stress), according to previous reports [37,38].

#### 2.5.2. Animals, Artificial Insemination, and Recording of Litter Traits

To evaluate reproductive performance, 35 boars (1–5 years old; 14 K, 12 DK, and 9 KD) and 647 sows (240 K, 261 DK, and 146 KD) were used. Moreover, the breeding stratagems were performed with K male × K female, DK male × DK female, and KD male × KD female, respectively.

Artificial insemination was performed using double inseminations. After mating, sows were housed individually and transferred to farrowing crates 7 days before the expected farrowing date. The farrowing rates, total number of piglets born and number of piglets born alive were recorded at farrowing to evaluate reproductive performance associated with boars and sows from different maternal origins. The sow population included both first-parity and multiparous animals, with parity ranging from 1 to 5.

### 2.6. Statistical Analysis

The percentage variables (sperm viability rates, acrosome integrity rates, mitochondrial activity rates, and farrowing rates) in this study were first transformed into arcsine square root to conform to the normal distribution, and then statistically analyzed by the Pro GLM of the SAS software package (version 9.4) [39]. Tukey’s HSD test was used to test whether the difference between groups reached a statistically significant level. The remaining variables (total piglets born and piglets born alive) were first tested for normality by the Proc Univariate of the SAS software package, and after confirming that the data collected in the field trial did not conform to the normal distribution, the Proc Npar1way in SAS was used for non-parameter test, and pairwise analysis was followed by the Dunn’s test.

## 3. Results

### 3.1. Differences in Sperm Viability Among Boars with Different Maternal Origins

Sperm viability of the three genetic groups (K, KD, and DK) under different incubation temperatures and durations is presented in Table 2. No significant differences were observed among K (85.5%), DK (81.1%), and KD (82.8%) boars in fresh semen (0 h). Under heat stress (42 °C), Meishan-derived cytoplasm conferred significantly higher (*p* < 0.05) sperm viability at 6 h (K and KD: 69.3% and 61.4% vs. DK: 47.3%) and 24 h (K and KD: 59.0% and 56.0% vs.DK: 47.4%) compared with Duroc-derived cytoplasm. At 12 h, K boars (73.5%) showed significantly greater (*p* < 0.05) viability than both DK (59.9%) and KD (64.7%). Similarly, under incubation at 39 °C (sow reproductive tract temperature), K and KD boars maintained significantly higher (*p* < 0.05) sperm viability than DK boars at all time points (6, 12, and 24 h), with values of 69.6%, 66.5%, and 66.6% in K and 62.5%, 65.5%, and 64.9% in KD, compared with 47.6%, 54.8%, and 51.0% in DK, respectively. In contrast, genotype effects were largely negligible under 17 °C storage, with significant differences detected only at 6 h, when K (86.8%) exceeded DK (79.5%) and KD (83.1%) (*p* < 0.05), whereas no differences were observed at 12 h (76.5–81.3%) or 24 h (79.1–84.0%). Across all genotypes, incubation at elevated temperatures (42 °C and 39 °C) significantly decreased sperm viability compared with 17 °C storage and fresh semen; however, the reduction was less pronounced in the K and KD groups than in the DK group (*p* < 0.05), suggesting enhanced thermotolerance associated with Meishan-derived maternal cytoplasm.

### 3.2. Acrosome and Plasma Membrane Integrity Among Boars with Different Maternal Origins

Acrosome and plasma membrane integrity of the three genetic groups under different incubation temperatures and durations are shown in Table 3. No significant differences were observed among K (75.1%), DK (69.2%), and KD (74.4%) boars in fresh semen (0 h). Under 42 °C incubation, acrosome integrity of semen was significantly higher (*p* < 0.05) in K and KD than in DK boars at 6 and 12 h (K: 60.5% and 61.9%; KD: 60.7% and 59.8% vs. DK: 44.0% and 49.5%). At 24 h, KD boars (57.8%) showed significantly greater (*p* < 0.05) acrosome integrity than DK (46.6%), but no significant differences were observed in K (53.7%) and DK. Similarly, under incubation at 39 °C, K and KD boars maintained significantly higher (*p* < 0.05) acrosome integrity than DK boars at 6 h (61.3% and 61.0% vs. 40.2%) and 24 h (57.1% and 61.0% vs. 47.4%). At 12 h, K boars (58.5%) showed significantly greater (*p* < 0.05) acrosome integrity than DK (43.8%), but no significant differences were observed in KD (55.9%) and DK. Under 17 °C storage, K and KD boars also showed significantly higher (*p* < 0.05) acrosome integrity than DK boars at 6 h (73.2% and 72.3% vs. 63.8%) and 12 h (73.4% and 68.7% vs. 62.3%). However, no significant differences (*p* > 0.05) were observed among genotypes at 24 h (66.5–71.8%). Overall, K and KD boars exhibited smaller reductions in acrosome integrity under heat stress, whereas genotype effects were minimal during 17 °C storage.

### 3.3. Mitochondrial Depolarization Among Boars with Different Maternal Origins

The proportion of sperm with mitochondrial depolarization under different incubation temperatures and durations is presented in Table 4. No significant differences were observed among K (17.0%), DK (19.9%), and KD (17.5%) boars in fresh semen (0 h). Under heat stress (42 °C), sperm from K and KD boars (Meishan-derived cytoplasm) exhibited significantly lower (*p* < 0.05) mitochondrial depolarization than DK boars at 6 h (31.8% and 32.8% vs. 41.8%) and 24 h (48.7% and 49.8% vs. 62.8%). However, no significant differences were detected among genotypes at 12 h (K: 43.3%; KD: 41.8%; DK: 49.5%). In contrast, no significant genotype differences were observed under 39 °C across 6–24 h, although numerical values ranged from 30.1% to 38.7% in K, 36.6% to 45.0% in DK, and 30.6% to 39.3% in KD. Similarly, under 17 °C storage, mitochondrial depolarization did not differ significantly among genotypes across 6–24 h (K: 17.9–24.0%; DK: 22.2–30.0%; KD: 19.5–28.8%). Overall, heat stress (42 °C and 39 °C) significantly increased mitochondrial depolarization in all groups, with the most pronounced effect observed at 42 °C in DK boars compared with K and KD boars. These findings were consistent with the patterns observed for sperm viability and acrosome integrity.

### 3.4. Environmental Conditions During Cool and Hot Seasons

Environmental data for the housing facility is shown in Table 5. During the hot season, the mean ambient temperature was 28.49 °C and the mean relative humidity was 83.75%, resulting in a mean THI of 81.02, corresponding to moderate heat stress. In contrast, during the cool season, the mean temperature was 22.18 °C and the mean relative humidity was 72.61%, with a mean THI of 69.84, indicating a thermally comfortable environment without heat stress. Overall, the hot season was characterized by substantially higher temperatures and THI values than the cool season. These distinct climatic conditions provided a clear environmental framework for evaluating seasonal effects on reproductive performance.

### 3.5. Reproductive Performance During Cool and Hot Seasons

Reproductive performance of the three genetic groups during the cool (December–March) and hot (June–September) seasons is presented in Table 6. During the hot season, farrowing rates of K (87.50%) and KD (85.90%) breed were significantly higher than that of DK breed (72.14%) (*p* < 0.05), whereas no significant differences were observed among groups during the cool season (K: 91.07%; DK: 84.29%; KD: 94.12%). When seasonal effects were examined within each genetic group, DK breed exhibited a significantly lower farrowing rate in the hot season compared with the cool season (72.14% vs. 84.29%, *p* < 0.05), whereas K and KD breed showed no significant seasonal decline. For litter size, during the hot season, K breed produced the largest litters (12.14 piglets), followed by KD (11.00 piglets) and DK (9.84 piglets), with K breed were significantly (*p* < 0.05) higher than that of DK breed. During the cool season, K breed (12.55 piglets) produced significantly larger litters than DK (10.47 piglets) (*p* < 0.05), whereas no significant difference was detected between DK and KD groups (11.19 piglets). Similarly, during the hot season, the number of live piglets was significantly higher in K (10.17 piglets) than in DK breed (8.20 piglets) (*p* < 0.05). During the cool season, K breed (10.53 piglets) produced more live piglets than DK (8.60 piglets) and KD groups (9.27 piglets) (*p* < 0.05), while no significant difference was observed between DK and KD groups. Collectively, genotype-related differences in reproductive performance were more pronounced during the hot season. Boars and sows carrying Meishan-derived maternal cytoplasm (K and KD) maintained a higher farrowing rate, litter size, and number of live piglets under heat stress conditions, whereas DK breed exhibited a significant seasonal decline, particularly in farrowing rate.

## 4. Discussion

Taiwan’s commercial pigs are predominantly derived from temperate-origin Western breeds. To enhance reproductive performance, Meishan pigs (Meishan, M) were introduced and crossed with Duroc pigs (Duroc, D) to develop KHAPS black pigs (KHAPS black pigs, K) [16]. However, with ongoing global warming, heat stress (HS) has become a critical challenge for the swine industry in tropical and subtropical regions such as Taiwan. It is well established that HS reduces reproductive performance in both boars [18,19,20] and sows [23,24]. Although Meishan pigs have been documented to exhibit superior reproductive performance [13,14], whether their thermotolerance is better than that of Western breeds remains unclear, and the potential contribution of maternal inheritance to thermotolerance in crossbred pigs has not been fully investigated. Therefore, the present study used K as the base population and applied the concept of maternal (cytoplasmic) inheritance to generate reciprocal crossbreds with Duroc, producing KD with M-derived cytoplasm and DK with D-derived cytoplasm. This design was used to evaluate semen quality following HS treatment and reproductive outcomes after artificial insemination (AI) during cool and hot seasons.

To support this evaluation, the maternal lineage of the experimental pigs within each breed was verified through pedigree records maintained by the breeding farm. When comparing semen from crossbred boars with different maternal origin under in vitro incubation at different temperatures, HS treatments (42 and 39 °C) significantly reduced sperm viability (Table 2) and acrosome integrity (Table 3), and significantly increased the proportion of mitochondrial depolarization (Table 4). In contrast, semen stored under a low-temperature condition (17 °C) was less affected. These findings are consistent with evidence that elevated temperature during semen delivery or holding can deteriorate boar sperm quality compared with low-temperature storage, accompanied by activation of apoptosis-related pathways [40]. Experimental exposure of boar spermatozoa to elevated temperatures significantly reduces mitochondrial function and sperm motility, partly through increased oxidative stress and disruption of mitochondrial regulatory pathways [18,33,34]. Increased ROS production promotes lipid peroxidation and oxidative damage to sperm membranes, leading to reduced membrane integrity and functional impairment [31,32]. In addition, heat stress–associated oxidative damage has been linked to sperm DNA damage and reduced fertilization potential, which may negatively affect early embryonic development [41]. More recent evidence further confirms that heat stress significantly decreases motion characteristics and membrane integrity in boar spermatozoa [40,41,42]. Collectively, these mechanisms may partially explain the decline in semen quality observed after HS treatment in this study.

Seasonal reproductive performance data further supported the adverse impact of heat exposure. In the present study, farrowing rates were lower in the hot season than in the cool season (Table 6), which is consistent with reports indicating that summer conditions are associated with reduced antioxidant capacity (e.g., TAC, GPx, and CAT) and increased lipid peroxidation (e.g., MDA), thereby impairing semen fertility under subtropical climates [42]. Taken together, increased oxidative stress and apoptosis-related pathways likely represent key mechanisms contributing to HS-induced declines in semen quality and reduced farrowing rate.

The cytoplasmic inheritance is maternally transmitted; K and KD pigs carry mtDNA that is 100% derived from Meishan pigs, whereas DK pigs carry mtDNA that is 100% derived from Duroc pigs. In contrast, the nuclear genetic composition differs among groups: K contains 50% D and 50% M nuclear inheritance, while DK and KD share the same nuclear genetic composition (75% D and 25% M) (Table 1). Under HS conditions (42 and 39 °C), semen from boars carrying Meishan-derived cytoplasm (K and KD) showed significantly better sperm viability, acrosome/membrane integrity, and mitochondrial depolarization profiles than semen from boars carrying Duroc-derived cytoplasm (DK) (Table 2, Table 3 and Table 4). Since the reproductive performance in this study was evaluated using mating between boars and sows of the same breed combination, the maternal lineage of both the boar and the sow may have contributed to the observed reproductive outcomes. Nevertheless, it is noteworthy that, under the same nuclear genetic composition (DK vs. KD; both 75% D and 25% M), the KD breed exhibited a significantly higher farrowing rate in the hot season than the DK breed, whereas no significant differences were observed in the cool season (Table 6). These results suggest that cytoplasmic inheritance may be associated with semen and embryo thermotolerance, and that Meishan-derived cytoplasm may confer greater resilience under heat stress. Although evidence on cytoplasmic effects on HS tolerance in pigs remains limited. However, in cattle, cytoplasmic origin has been implicated as a key determinant of thermotolerance. Embryos derived from heat-tolerant maternal backgrounds (cytoplasm) show enhanced resistance to heat shock, supporting a strong maternal contribution to thermotolerance [25,43]. Furthermore, studies involving somatic cell nuclear transfer (SCNT) and related approaches indicate that when cytoplasm originates from thermotolerant cattle breeds, cells and embryos can exhibit improved survival and developmental competence following heat challenge, even when the nuclear background is controlled [29,30,44]. These findings suggest that cytoplasm-associated mechanisms—potentially related to mitochondrial functional stability and oxidative stress regulation may influence physiological resilience under HS. Nevertheless, species-specific differences should be considered, and similar mechanisms require further verification in pigs.

In addition to cytoplasmic origin, maternal environment during early development and potential Mito–nuclear interactions may also influence thermotolerance. Moreover, because the field mating design in this study used boars and sows of the same group, farrowing rate and litter size may reflect combined boar and sow effects. Previous work has shown that mtDNA haplogroups can be associated with litter size differences in pigs, indicating that maternal lineages may contribute to reproductive performance [45]. Additionally, Meishan pigs have been reported to show earlier conceptus elongation, more uniform conceptus development, and higher embryonic survival during early embryonic development, which may contribute to improved prolificacy [11]. Accordingly, the superior farrowing rate and litter size observed for KD compared with DK in the hot season (Table 6) may be positively associated with Meishan-derived cytoplasm, although combined male and female effects cannot be excluded in the present design.

The present results also showed that K had a consistently greater total number of piglets born than KD in both seasons (Table 6), whereas the farrowing rate was comparable between the two groups. Although both K and KD share Meishan-derived cytoplasm, K has a higher proportion of Meishan nuclear inheritance (50% M) than KD (25% M), suggesting that Meishan-derived nuclear genetics also contribute to improved reproductive outcomes. This interpretation is consistent with reports of both direct and maternal genetic effects on litter traits in black pig populations [16]. In addition, it has been suggested that maternal-line effects on litter traits can be modulated by nuclear background or breed combinations [10], supporting the possibility of cytoplasmic-by-nuclear interactions affecting reproductive performance.

Since previous studies have demonstrated that heat stress impairs mitochondrial function in boar spermatozoa, this aspect was not examined in the present study. In the future, investigating the effects of heat stress on sperm mitochondrial function in boars with different maternal origin may provide further insight into the relationship between maternal origin and thermotolerance. Overall, HS treatments (42 and 39 °C) impaired boar semen quality; however, semen from boars carrying Meishan-derived cytoplasm (K and KD) was less negatively affected than that from boars carrying Duroc-derived cytoplasm (DK). Consistently, under hot-season mating, K and KD exhibited a higher farrowing rate and litter size than DK. Collectively, these in vitro and seasonal reproductive outcomes suggest that boars and sows carrying Meishan-derived cytoplasm maintain greater physiological stability and reproductive performance under heat stress, although the molecular mechanisms remain to be elucidated.

## 5. Conclusions

This study demonstrated that heat stress (42 and 39 °C by incubation method) significantly reduced sperm viability, acrosome integrity, and plasma membrane integrity across all genetic groups. However, boars carrying Meishan-derived maternal cytoplasm (K and KD) consistently exhibited smaller declines in semen quality under elevated temperature conditions than those carrying Duroc-derived cytoplasm (DK). Field reproductive data further supported these findings, as K and KD breeds maintained higher farrowing rates during the hot season, whereas genotype differences were minimal during the cool season.

The reciprocal cross design allowed partial separation of cytoplasmic and nuclear genetic effects. The results indicate that maternal inheritance may contribute to thermotolerance and reproductive performance under heat stress in pigs. These findings highlight the potential importance of maternal lineage in breeding strategies aimed at improving heat tolerance in swine production systems in tropical and sub-tropical regions under ongoing climate change.

## Figures and Tables

**Table 1:** Breeding scheme and genetic composition of boars with different maternal origins.

Boar Breed	Breeding Mode		Genetic Inheritance, %			
			Cytoplasm		Nuclear	
	Dam	Sire	M	D	M	D
M	M	M	100	0	100	0
D	D	D	0	100	0	100
K	M	D	100	0	50	50
KD	K	D	100	0	25	75
DK	D	K	0	100	25	75

M: Meishan; K: M female mated with D male; KD: K female mated with D male; DK: D female mated with K male.

**Table 2:** Sperm viability of boars with different maternal origins after heat stress.

Treatment Temperature	Incubation	Breed		
		K	DK	KD
37 °C	0 h (fresh)	85.5 ± 1.2 ^a, A^	81.1 ± 1.9 ^a, A^	82.8 ± 1.7 ^a, A^
42 °C	6 h	69.3 ± 3.2 ^a, CD^	47.3 ± 4.7 ^b, B^	61.4 ± 3.6 ^a, B^
	12 h	73.5 ± 1.8 ^a, BC^	59.9 ± 2.5 ^b, B^	64.7 ± 2.3 ^b, B^
	24 h	59.0 ± 2.5 ^a, D^	47.4 ± 2.5 ^b, B^	56.0 ± 2.3 ^a, B^
39 °C	6 h	69.6 ± 3.6 ^a, CD^	47.6 ± 4.6 ^b, B^	62.5 ± 3.4 ^a, B^
	12 h	66.5 ± 2.5 ^a, CD^	54.8 ± 3.5 ^b, B^	65.5 ± 2.2 ^a, B^
	24 h	66.6 ± 2.4 ^a, CD^	51.0 ± 3.4 ^b, B^	64.9 ± 2.9 ^a, B^
17 °C	6 h	86.8 ± 1.1 ^a, A^	79.5 ± 2.6 ^b, A^	83.1 ± 1.4 ^ab, A^
	12 h	81.3 ± 4.6 ^a, AB^	76.5 ± 3.5 ^a, A^	80.8 ± 2.4 ^a, A^
	24 h	84.0 ± 2.5 ^a, AB^	79.1 ± 2.0 ^a, A^	83.5 ± 1.1 ^a, A^

Each breed included four boars, and each boar was repeated three times in each treatment. Data are presented as mean ± SEM. ^A–D^ When comparing within the same column, the means with different superscripts differ at *p* < 0.05. ^a,b^ When comparing within the same row, the means with different superscripts differ at *p* < 0.05.

**Table 3:** Sperm acrosome integrity of boars with different maternal origins after heat stress.

Treatment Temperature	Incubation	Breed		
		K	DK	KD
37 °C	0 h (fresh)	75.1 ± 2.6 ^a, A^	69.2 ± 2.0 ^a, A^	74.4 ± 2.1 ^a, A^
42 °C	6 h	60.5 ± 2.2 ^a, C^	44.0 ± 3.1 ^b, B^	60.7 ± 4.3 ^a, ABC^
	12 h	61.9 ± 1.7 ^a, BC^	49.5 ± 2.3 ^b, B^	59.8 ± 2.3 ^a, ABC^
	24 h	53.7 ± 2.3 ^ab, C^	46.6 ± 2.6 ^b, B^	57.8 ± 2.1 ^a, BC^
39 °C	6 h	61.3 ± 2.6 ^a, C^	40.2 ± 3.5 ^b, B^	61.0 ± 4.7 ^a, ABC^
	12 h	58.5 ± 3.4 ^a, C^	43.8 ± 2.5 ^b, B^	55.9 ± 4.2 ^ab, C^
	24 h	57.1 ± 1.6 ^a, C^	47.4 ± 2.4 ^b, B^	61.0 ± 2.2 ^a, ABC^
17 °C	6 h	73.2 ± 1.4 ^a, A^	63.8 ± 2.9 ^b, A^	72.3 ± 2.4 ^a, AB^
	12 h	73.4 ± 1.4 ^a, A^	62.3 ± 2.3 ^b, A^	68.7 ± 4.7 ^ab, ABC^
	24 h	71.8 ± 1.5 ^a, AB^	66.5 ± 2.5 ^a, A^	71.2 ± 1.7 ^a, AB^

Each breed included four boars, and each boar was repeated three times in each treatment. Data are presented as mean ± SEM. ^A–C^ When comparing within the same column, the means with different superscripts differ at *p* < 0.05. ^a,b^ When comparing within the same row, the means with different superscripts differ at *p* < 0.05.

**Table 4:** Sperm mitochondrial depolarization of boars with different maternal origins after heat stress.

Treatment Temperature	Incubation	Breed		
		K	DK	KD
37 °C	0 h (fresh)	17.0 ± 1.6 ^a, E^	19.9 ± 2.2 ^a, E^	17.5 ± 2.0 ^a, F^
42 °C	6 h	31.8 ± 1.7 ^b, CD^	41.8 ± 2.8 ^a, BC^	32.8 ± 3.1 ^b, BC^
	12 h	43.3 ± 2.4 ^a, AB^	49.5 ± 3.6 ^a, B^	41.8 ± 1.6 ^a, AB^
	24 h	48.7 ± 2.3 ^b, A^	62.8 ± 3.7 ^a, A^	49.8 ± 2.3 ^b, A^
39 °C	6 h	30.1 ± 2.4 ^a, CD^	36.6 ± 2.0 ^a, BCD^	30.6 ± 2.5 ^a, BCD^
	12 h	34.3 ± 2.5 ^a, BC^	40.7 ± 2.7 ^a, BC^	39.3 ± 3.6 ^a, ABC^
	24 h	38.7 ± 2.0 ^a, ABC^	45.0 ± 2.6 ^a, B^	38.4 ± 2.3 ^a, ABC^
17 °C	6 h	17.9 ± 1.3 ^a, E^	22.2 ± 2.4 ^a, E^	19.5 ± 1.4 ^a, EF^
	12 h	23.6 ± 2.2 ^a, DE^	27.4 ± 2.6 ^a, DE^	27.3 ± 2.1 ^a, DEF^
	24 h	24.0 ± 2.0 ^a, DE^	30.0 ± 2.9 ^a, CDE^	28.8 ± 1.9 ^a, CDE^

Each breed included four boars, and each boar was repeated three times in each treatment. Data are presented as mean ± SEM. ^A–F^ When comparing within the same column, the means with different superscripts differ at *p* < 0.05. ^a,b^ When comparing within the same row, the means with different superscripts differ at *p* < 0.05.

**Table 5:** Average temperature, humidity, and THI from June 2019 to March 2024.

Season	Month	Temp (°C)	RH (%)	THI
Hot	June–Sep	28.49 ± 0.13 ^a^	83.75 ± 0.68 ^a^	81.02 ± 0.24 ^a^
Cool	Dec–March	22.18 ± 0.30 ^b^	72.61 ± 0.90 ^b^	69.84 ± 0.47 ^b^

Data are presented as mean ± SEM. ^a,b^ Within a column, means without a common superscript differ (*p* < 0.05).

**Table 6:** Reproductive performance of boars with different maternal origins during cool and hot seasons.

Item	Breed		
	K	DK	KD
**Hot season (June–September)**			
Number of matings	128	140	78
Number of farrowing sows (*n* = first-parity sows)	112 (44)	101 (30)	67 (16)
Farrowing rate (%)	87.50 ± 2.93 ^a, A^	72.14 ± 3.80 ^b, B^	85.90 ± 3.97 ^a, A^
Total piglets born	12.14 ± 0.38 ^a, A^	9.84 ± 0.33 ^b, A^	11.00 ± 0.32 ^ab, A^
Piglets born alive	10.17 ± 0.33 ^a, A^	8.20 ± 0.32 ^b, A^	9.39 ± 0.32 ^ab, A^
**Cool season (December–March)**			
Number of matings	112	121	68
Number of farrowing sows (*n* = first-parity sows)	101 (21)	102 (23)	64 (14)
Farrowing rate (%)	91.07 ± 2.71 ^a, A^	84.29 ± 3.32 ^a, A^	94.12 ± 2.87 ^a, A^
Total piglets born	12.55 ± 0.38 ^a, A^	10.47 ± 0.31 ^b, A^	11.19 ± 0.4 ^ab, A^
Piglets born alive	10.53 ± 0.34 ^a, A^	8.60 ± 0.30 ^b, A^	9.27 ± 0.38 ^b, A^

Data are presented as mean ± SEM. ^A,B^ Within a column, means without a common superscript differed between hot and cool seasons in the same measure item (*p* < 0.05). ^a,b^ Within a row, means without a common superscript differed (*p* < 0.05).

## Data Availability

Data are available only upon agreement with Taiwan Livestock Research Institute and should be requested directly from the authors.
